# The SAGES MASTERS program presents: the 10 seminal articles for the Laparoscopic Right Colectomy Pathway

**DOI:** 10.1007/s00464-022-09310-x

**Published:** 2022-05-18

**Authors:** Deborah S. Keller, Giovanni Dapri, Alexis L. Grucela, George Melich, Ian M. Paquette, Virginia O. Shaffer, Konstantin Umanskiy, Angela H. Kuhnen, Jeremy Lipman, Elisabeth C. Mclemore, Mark Whiteford, Patricia Sylla, the Colorectal Committee Right Colon Subcommittee

**Affiliations:** 1 Division of Colon and Rectal Surgery, Department of Surgery, University of California Davis Medical Center, Sacramento, CA USA; 2 International School of Reduced Scar Laparoscopy, Brussels, Belgium; 3 Division of Colon and Rectal Surgery, Northern Westchester Hospital, Mount Kisco, NY USA; 4 Department of General Surgery, Royal Columbian Hospital, New Westminster, BC Canada; 5 Department of Surgery, University of Cincinnati School of Medicine, Cincinnati, OH USA; 6 Department of Surgery, Emory University, Atlanta, GA USA; 7 Department of Surgery, The University of Chicago Pritzker School of Medicine, Chicago, IL USA; 8 Division of Colon and Rectal Surgery, Lahey Clinic, Burlington, MA USA; 9 Department of Surgery, Cleveland Clinic Foundation, Cleveland, OH USA; 10 Department of Surgery, Colorectal Surgery, Kaiser Permanente Los Angeles Medical Center, Los Angeles, CA USA; 11 Oregon Clinic and Providence Cancer Centre, Portland, OR USA; 12 Division of Colon and Rectal Surgery, Icahn School of Medicine at Mount Sinai, New York, NY USA

**Keywords:** SAGES Masters program, Laparoscopic colorectal surgery, Laparoscopic right colectomy, Seminal article, Colon cancer, Complete mesocolic excision (CME)

## Abstract

**Background:**

As one of the 12 clinical pathways of the Society of American Gastrointestinal and Endoscopic Surgeons (SAGES) Masters Program, the Colorectal Pathway intends to deliver didactic content organized along 3 levels of performance (competency, proficiency and mastery) each represented by an anchoring procedure (laparoscopic right colectomy, laparoscopic left/sigmoid colectomy, and intracorporeal anastomosis during minimally invasive (MIS) ileocecal or right colon resection). In this article, the SAGES Colorectal Task Force presents focused summaries of the top 10 seminal articles selected for laparoscopic right colectomy which surgeons should be familiar with.

**Methods:**

Using a systematic literature search of Web of Science, the most cited articles on laparoscopic right colectomy were identified, reviewed, and ranked by the SAGES Colorectal Task Force and invited subject experts. Additional articles not identified in the literature search were included if deemed impactful by expert consensus. The top 10 ranked articles were then summarized, with emphasis on relevance and impact in the field, findings, strengths and limitations, and conclusions.

**Results:**

The top 10 seminal articles selected for the laparoscopic right colectomy anchoring procedure include articles on surgical techniques for benign and malignant disease, with anatomical and video illustrations, comparative outcomes of laparoscopic vs open colectomy, variations in technique with impact on clinical outcomes, and assessment of the learning curve.

**Conclusions:**

The top 10 seminal articles selected for laparoscopic right colectomy illustrate the diversity both in content and format of the educational curriculum of the SAGES Masters Program to support practicing surgeon progression to mastery within the Colorectal Pathway.

Surgical competence is a complex, multidimensional process that takes time and training. There is an undisputed need for learners to build a framework of basic skills before carrying out surgical procedures on patients [1]. A great deal of attention has been placed on the competency of trainees, as there has been a shift in the style of surgical education over the last decade, where trainees spend less time in the hospital, in the operating room, and with mentors from duty-hour restrictions [2, 3]. However, there is the same need for practicing surgeons to achieve and maintain competence, especially in the acquisition of new skills. Learning a new technique and gaining competence, proficiency, and mastery are even more challenging for practicing surgeons due to time and financial constraints limiting the ability to take time off to participate in educational events.

Recognizing the need for practicing surgeons to stay current with evidenced-based new technologies, new procedures, and safely implement them into clinical practice, the Society of American Gastrointestinal and Endoscopic Surgeons (SAGES) formed the Masters Program, a structured curriculum for deliberate, lifelong learning. This educational curriculum incorporates SAGES educational materials and guidelines, social media content, coaching, and mentoring to facilitate the learning of surgeons in practice [4]. As one of the 12 SAGES Masters Program clinical pathways, the Colorectal Pathway will deliver curricula centered around three anchoring procedures, laparoscopic right colectomy, laparoscopic left/sigmoid colectomy, and intracorporeal anastomosis during MIS right-sided resections. The educational content will be organized along three levels of performance (competency, proficiency, and mastery) to facilitate assessment of the learner’s fund of knowledge, clinical management and decision-making skills, and technical skills.

Members of the SAGES Colorectal Task Force and subject experts were recently tasked with selecting the top 10 seminal articles for each anchoring procedure of the relevant clinical pathway, based on a systematic literature search of Web of Science of the most cited and/or most relevant articles by expert consensus [5]. The rationale of this effort was to identify the most impactful articles with respect to teaching the anchoring procedure, with focus on surgical technique, clinical effectiveness, and outcomes [5]. The present manuscript summarizes the content of the 10 seminal articles selected for the Colorectal Pathway Laparoscopic Right Colectomy Anchoring Procedure, in order to educate SAGES members on the clinical considerations, technical steps, clinical outcomes, and tips to safely implement this procedure into practice.

## Materials and methods

A systematic literature review was performed in December 2020 and updated in January 2022 for all articles published on the topic of laparoscopic right colectomy. Using the Web of Science, Google Scholar, Altmetric Index (for articles published from 2011 onward), and frequency of citations, the top 30 articles with the highest citation index were selected, reviewed, and ranked by the members of the SAGES Colorectal Task Force as well as by invited subject experts [5]. Reviewers were allowed to suggest additional articles not identified in the search if deemed impactful in the teaching, training, and/or safe adoption of laparoscopic right colectomy. Reviewers were then asked to rank articles taking into consideration citation index as well as relevance in the field, impact on education, and adoption of laparoscopic right colectomy. Among 30 experts invited to review the articles, 28 responses were received, and consensus was achieved with the top 10 seminal articles for each of the Masters Colorectal pathway anchoring procedure [6–15]. The top 10 seminal articles were then reviewed by the members of the Colorectal Task Force Right Colon Subcommittee, summaries were compiled and presented here by addressing the following questions: 1. Why is this a top 10 article? 2. What is unique about this paper? 3. Why is it important to read this paper before you do the relevant procedure? 4. What has been the impact of this paper in the field? 5. What are the study findings? 6. What are the strengths and limitations of paper, and 7. What are the conclusions of this article?

## Results

The citation index for the top 10 seminal articles selected for the Colorectal Pathway laparoscopic right colectomy anchoring procedure ranged from 1.8 to 47.5 on Google Scholar, from 1.0 to 33.4 on Web of Science, and Altmetric attention scores in the 72.0% to 97.0% percentiles (Table 1). Articles in the top 10 list include description of procedural steps and video demonstration of laparoscopic and robotic right colectomy for benign disease and oncologic resection for cancer, variations in techniques, clinical outcomes, and assessment of the learning curve. They are presented here in order of 1 to 10 per the reviewers’ rank, with 1 being the highest ranked.

**Table 1:** Citation metrics for the top 10 right colectomy articles (search performed January 2022)

Rank	Author	Year	Web of science	Google Scholar	Times cited	Altmetric Attention Score	Altmetric percentile
1	Rondelli	2012	5.16	8.7	59	NA	NA
2	Dijkstra	2015	1.66	3.0	29	27	94%
3	Liang	2007	2.6	4.4	35	NA	NA
4	Rickard	2017	3.0	4.5	3	17	90%
5	Di Buono	2021	1.0	3.0	5	9	88%
6	Strey	2018	3.0	4.2	27	89	97%
7	Lee	2016	2.5	3.5	32	5	72%
8	Cabot	2010	2.5	4.6	54	NA	NA
9	van Oostendorp	2012	1.8	5.0	95	19	90%
10	Tekkis	2005	33.38	47.5	799	NA	NA

### Rondelli et al (2012) Is laparoscopic right colectomy more effective than open resection? A meta-analysis of randomized and nonrandomized studies [6]

This is comprehensive meta-analysis of available studies at the time of publication (2012) comparing the outcomes of laparoscopic and/or laparoscopic-assisted right colectomy (LRC) with open right colectomy (ORC). The authors aimed to answer the question of whether laparoscopic right colectomy provided definitive benefits relative to the open approach, in light of the fact that prior to this publication, several authors speculated that laparoscopic right colectomy may not have the same advantages as laparoscopic resection elsewhere in the colon.

The review includes randomized and non-randomized studies as well as patients with benign and malignant diseases. Overall, 17 studies comprising 1489 patients were included in the analysis—710 (47.7%) LRC and 779 (52.3%) ORC. The authors performed a subgroup analysis of patients undergoing right colectomy for malignant diseases. The outcome measures used to compare LRC to ORC were the following: intraoperative (operating time and estimated blood loss), postoperative recovery (time to suspension of postoperative analgesia, time to start normal diet, time to first bowel movement and length of hospital stay), early postoperative (anastomotic leakage, urinary infection, wound infection, chest infection, prolonged postoperative ileus, overall morbidity, overall mortality, intraabdominal bleeding, deep-vein thrombosis and reoperation rate), intraoperative oncological (mean number of dissected lymph nodes and mean length of the specimen), and long-term oncological (local recurrence, port-site/incision recurrence, and distant recurrence).

The mean operative time was longer in the group of patients undergoing LRC [weighted mean difference (WMD) = 37.94, 95% CI 25.01 to 50.88; *P* < 0.00001]. Intraoperative blood loss was in favor of LRC (WMD − 96.61; 95% CI − 150.68 to − 42.54; *P* = 0.0005). Length of hospital stay was in favor of LRC (WMD − 2.29; 95% CI − 3.96 to − 0.63; *P* = 0.007). Short-term postoperative morbidity was less in LRC group (OR 0.64; 95% CI 0.49 to 0.83; *P* = 0.0009).

Even though this meta-analysis has some limitations including the fact that the results are mostly based on non-randomized studies with heterogeneous data and surgeons of varying experience, the results suggest that LRC has benefits compared to ORC (shorter hospital stay, less intraoperative blood loss, and less short-term overall morbidity), accompanied by comparable oncological outcomes (number of harvested lymph nodes, local and distant recurrence). This publication served as validation and helped support adoption of laparoscopic right colectomy for benign and malignant indications.

Overall, this is a well-designed meta-analysis that specifically looks at outcomes of laparoscopic versus open right hemicolectomy. Despite longer operative times, the benefits of shorter hospital stay, lower intraoperative blood loss, and lower short-term overall morbidity support the notion that laparoscopic right colectomy is superior to conventional open technique.

This paper was published several years after the COST trial was completed [16]. It reviews the papers published during the paradigm shift of wide acceptance of laparoscopic colectomy as a safe, feasible, and preferred technique for both benign and malignant diseases of the colon.

In many ways, this study confirms the findings of the COST trial, but focused its evaluation only on the right colectomy procedure. It is important as it confirms that the benefits identified by the COST trial, the initial randomized controlled trial on the topic, are applicable and generalizable when laparoscopic right colectomy is used by the surgical community.

Using the power of a meta-analysis, this paper confirmed the notion that outcome of right hemicolectomy performed laparoscopically or with laparoscopic assistance are superior in several aspects to those of open approach.

### Dijkstra FA et al (2015) Procedural key steps in laparoscopic colorectal surgery, consensus through Delphi methodology [7]

This manuscript describes the key procedural steps for laparoscopic right colectomy and sigmoid colectomy that were determined and agreed upon by expert consensus. Moreover, these key steps were defined to serve as the basis for a video-assisted teaching curriculum. While several procedural training curricula in laparoscopic colorectal surgery have been validated and published, none have focused on dividing surgical procedures into well-identified segments, which can be trained and assessed separately.

Rigorous Delphi methodology was employed by an expert panel of 22 surgeons from the Netherlands to identify 25 key essential procedural steps for laparoscopic right colectomy and 24 essential procedural steps for laparoscopic sigmoid colectomy.

The Delphi method was used to develop a consensus on key steps of both laparoscopic procedures. A list of 31 steps for laparoscopic right hemicolectomy and 37 steps for laparoscopic sigmoid colectomy was compiled from textbooks and national and international guidelines. Using an online questionnaire, 22 experts from 12 hospitals within the teaching region (Groningen University, The Netherlands) were invited to rate all steps on a Likert scale on importance for the procedure.

Consensus was reached after two rounds among 16 participating experts. Of these, 14 (88%) completed the questionnaire for both procedures. Cronbach’s alpha was 0.79 for laparoscopic right hemicolectomy and 0.91 for laparoscopic sigmoid colectomy, showing high internal consistency between the experts. For laparoscopic right hemicolectomy, 25 key steps were established; for laparoscopic sigmoid colectomy, 24 key steps were established.

The aim of the study was to use the identified key steps to create Intraoperative Video-Enhanced Surgical procedure Training (INVEST) videos for both procedures. Eventually, the goal was to create and validate a procedure-specific assessment tool, suitable for incorporation in the training and certification process of gastrointestinal surgeons.

Overall, this article informs surgeons on the critical procedural steps of a laparoscopic right hemicolectomy and laparoscopic sigmoid colectomy, with the goal of developing universal training videos. This manuscript provides expert-based consensus on the essential procedural steps of laparoscopic right hemicolectomy and provides a framework for training and assessing procedural competency in this procedure. This manuscript serves as the basis for a standardized transferrable and efficient video-assisted training curriculum for core laparoscopic colorectal procedures.

### Liang JT et al (2007) Laparoscopic medial-to-lateral approach for the curative resection of right-sided colon cancer [8]

This manuscript provides a succinct, multimedia description of an organized approach to laparoscopic medial-to-lateral approach for right colon cancer. In addition to providing clinical outcomes, it includes a step-by-step illustrative video of the critical portions of the operation.

This paper is one of the first to describe the medial-to-lateral approach to right hemicolectomy for colon cancer and provides oncologic results in support of this approach. The report provides surgeons guidance on how to approach the operation and the key steps involved. It is valuable and educational for the surgeon reader/viewer, as the steps are well described and illustrated.

In addition to providing procedural guidance on how to safely perform laparoscopic right hemicolectomy for colon cancer using the medial-to-lateral approach, oncologic data with this approach are also provided to benchmark results of surgeons who are early along their learning curve.

For surgeons along their learning curve, this manuscript provides detailed guidance on the procedural steps and extent of lymph node dissection required during the laparoscopic radical resection of right-sided colon cancer. The medial-to-lateral approach provides a clear and reproducible framework that can be safely adopted, with acceptable clinical outcomes.

The authors have previously evaluated the lateral-to-medial approach for resection of rectosigmoid cancer and shown that the medial-to-lateral approach reduced operative time and the postoperative pro-inflammatory response. This study’s aim was to further examine the feasibility and outcomes of the medial-to-lateral laparoscopic approach for the curative resection of right-sided colon cancer. The authors hypothesized that the oncologic advantages of an early vessel division and a “no-touch” technique, in addition to preserving the lateral abdominal wall attachments of the colon, would result in better exposure, facilitating dissection.

The study reviewed outcomes of a consecutive case series of 104 patients who underwent curative laparoscopic right colectomy with a medial-to-lateral approach for stage II and III right-sided colon cancer over a 5-year period. The procedural steps are described and a supplementary video is included.

Laparoscopic procedures were completed with a 5.7% rate of intraoperative complications, 1.9% leak rate, and 3.8% incidence of surgical site infection. The lymph node yield was 16 ± 2.8 nodes.

The manuscript includes a detailed description of the laparoscopic medial-to-lateral approach for right colon cancer and is supplemented by a procedural video which serves as an operative guide to surgeons along their learning curve. Limitations of the manuscript relate to the small size of the retrospective cohort included, and the lack of long-term oncologic data.

This multimedia manuscript serves as a useful guide for surgeons adopting the laparoscopic medial-to-lateral approach for right colon cancer. This manuscript supports the feasibility and safety of the laparoscopic approach for the curative resection of right-sided colon cancer and also demonstrates technical steps to assist surgeons along the learning curve in the safe adoption of this approach.

### Rickard MJFX et al (2017) Three steps and a join: a simple guide to right- and left-sided medial-to-lateral laparoscopic colorectal surgery [9]

This article describes a simple and reliable technique of performing a laparoscopic right hemicolectomy, which has proven safe, effective, and durable in the authors’ experience with teaching this core procedure.

The manuscript and instructional video provide with a simple strategy to complete medial-to-lateral dissection during laparoscopic right colectomy, with a focus on safe technical steps that can be used during teaching of trainees.

This article will serve to pre-train the surgeon to break down the procedure into clear steps, so they can progress through the procedure efficiently.

This article supports the notion that laparoscopic right colectomy can be divided into simple and reproducible steps that can be taught and safely performed by trainees. Data presented include the anastomotic leak rate following laparoscopic right colectomy performed using this approach from a relatively large case series. High-quality video of the procedure is appended to the paper as supplementary material.

Various approaches and techniques have been described for laparoscopic right hemicolectomy. The three main approaches include (1) the lateral-to-medial approach widely known from standard open surgery; (2) the top-down approach where the lesser sac is entered first and the colon is dissected from retroperitoneum in retrograde fashion; and (3) the medial-to-lateral approach developed specifically for laparoscopic surgery. With all these variations, few works have standardized the procedure into simple steps and demonstrated the feasibility of using these steps for training. The goal of the article was to divide the medial-to-lateral approach into three well-defined steps plus completion of an anastomosis and report short-term outcomes of this approach in a large series with active participation of trainees.

A total of 543 consecutive laparoscopic right hemicolectomy cases were performed from 2006 to 2016 in a teaching hospital. Technical steps included placement of a 12 mm umbilical Hasson port and three additional 5 mm ports in the suprapubic, left lateral, and subxiphoid positions. The steps included the following: (1) medial-to-lateral dissection of the mesentery under the ileocolic vessels and division of the ileocolic vessels high at their origin; (2) mobilization of the hepatic flexure through dissection into the lesser sac, incising the peritoneum over the transverse colon, and then continuing dissection around the hepatic flexure into the right line of Toldt in a retrograde fashion to the lower margin of the cecum; (3) mobilization of the terminal ileum by retracting the ileocolic junction cranially and dissecting under the mesentery of the terminal ileum to complete the dissection; and (4) completion of the anastomosis (referred to by authors as the join), which is performed extracorporeally. Additional guidance is provided to overcome technical difficulties such as operating for active Crohn’s disease. A clearly narrated and expertly edited instructional video of all the key steps was created as a further tool for learning the procedure.

In this series of 543 cases, clinically significant leak rate occurred in 0.6% (3/543). Trainees performed a large proportion of the procedures. Procedures were performed by surgical trainees in a supervised environment without any statistical difference in complication rates between trainees and surgeons.

The strength of this work lies primarily in its simple design and potential for widespread dissemination. The major limitation consists in the lack of oncological considerations in malignant disease, including long-term oncological outcomes especially in locally advanced disease. Another unaddressed issue is the potential added value of performing intracorporal rather than extracorporeal anastomosis.

Overall, this report with its instructional video can serve as an invaluable tool in understanding, teaching, and performing laparoscopic right hemicolectomy for benign disease.

### Di Buono G et al (2021) Feasibility and safety of laparoscopic CME for right-sided colon cancer: short-term outcomes. A randomized clinical study [10]

CME with central vascular ligation (CVL) was proposed in 2009 Hohenberger et al. to address recurrences in right-sided colon cancer resections. The procedure routinely identifies the ileocolic vessels followed by ligation at the origin from the superior mesenteric vessels, dissection of the Toldt fascia between the visceral peritoneum of the ascending colon and the Gerota fascia, performed in a medial-to-lateral and bottom-up dissection, removal of the lymph nodes and adipose tissue covering the duodenum and the head of the pancreas, detachment of the mesocolon, and ligation of the origin of the right colic vessels, gastrocolic trunk of Henle, and right branch of the middle colic vessels. Concerns of the safety, feasibility, and oncologic superiority have limited widespread implementation of the CME in right colon cancer resections. Several case series and cohort studies have been published, claiming safety and feasibility of the procedure. However, this work represents the first controlled trial evaluating this approach for right-sided colon cancer.

This work is a top 10 article because it uses a randomized controlled design to evaluate the feasibility and safety of laparoscopic CME in right colonic resection within 30 days of surgery (short-term outcome). The authors randomized 67 patients to resection with and 65 patients without CME between 2015 and 2019 with procedures performed by 1 of 2 surgeons experienced in colorectal resections and laparoscopic CME in a high-volume center. CME procedures were performed as detailed above while the non-CME group had no exposure of the superior mesenteric vessels and no dissection of the pre-duodeno-pancreatic tissue. This paper is unique because it evaluated primary endpoints for safety and feasibility, such as operative time, intraoperative blood loss, and conversion rate, as well as postoperative complications and histopathologic data for oncological outcomes. It is important to read this paper prior to implementing CME because objective measures of high-quality CME, such as the fusion fascia of Fredet, are discussed.

This paper has a major impact in the field by demonstrating that objective measures of high-quality CME are directly related to short-term oncological results. The CME group had a significantly longer mean operative time than the non-CME group (216.3 min vs 191.5 min, *P* = 0.005), but also a higher number of lymph nodes (23.8 vs 16.6, *P* < 0.001) and larger surgical specimens (34.3 cm vs 29.3 cm, *P* = 0.002). There were no significant differences in intraoperative blood loss, conversion rate, leakage, or other postoperative complications. No conversion was linked to difficult mesocolic dissection or vascular injuries. Further, the complications seen in the CME group were due to adhesions and had no relationship with the CME.

The study has several strengths, starting with the randomized design; correct randomization is crucial for an accurate result analysis. It was powered to demonstrate a clinically acceptable 30-min difference in operative time and a clinically acceptable difference in the number of lymph nodes between the groups, and the study recruited to appropriate power. In addition, the authors used videos to objectively review and confirm the dissection was performed precisely per standardized description. Limitations in the study were also appreciated. It was a single-center study with only 2 surgeons performing CME procedures, so there could be bias in preoperative characteristics and lack of generalizability. Second, they only recorded the short-term oncologic and postoperative outcomes, so any complications after 30 days and long-term oncologic outcomes are not available. Finally, they did not measure the length of the vascular pedicle or the area of the resected mesentery, variables specific to CME that can be used as quality metrics.

In conclusion, this well-performed study found that in experienced laparoscopic surgeons, a laparoscopic CME and CVL is a safe and feasible with greater a lymph node harvest and no significant increase in complications. Multicenter randomized controlled trials with a larger sample size and long-term follow will help validate the long-term oncological results of laparoscopic CME for right-sided colon cancer.

### Strey CW et al (2018) Laparoscopic right hemicolectomy with complete mesocolic excision (CME): standardization using the "critical view" concept [11]

With the data supporting oncologic advantages of right hemicolectomy with CME for colon cancer, routine implementation into practice has been advocated. In-depth training of colorectal surgeons for safe implementation of this method is advised, as was done for total mesocolic excision (TME) for rectal cancer. Prior to implementing procedure-related training, agreement on the relevant surgical anatomy and standardization of the steps of the operation are needed. In this work, the authors commence a multidisciplinary working group to create a step-wise framework for education. The group aimed to establish a consensus of steps for a standardized laparoscopic right hemicolectomy that meets all CME criteria, with maximal surgical safety.

A group of 13 expert laparoscopic colorectal surgeons, where each had a minimum of 13 years of post-certification experience and had performed at least 750 colorectal resections, more than 500 laparoscopic colorectal resections and more than 90 laparoscopic right hemicolectomies, one anatomist, and one graphic artist met to define the requirements of a safe, teachable, and radical CME. They achieved consensus that the procedure needed a clear definition of surgical anatomy and oncologic radicality and description of procedure-related hazards. They also proposed clear depictions of the anatomy using the “open-book” model of the fascial and vascular relations, where “pages” of the book represent the embryologically defined anatomical layers. They included a critical view of safety in each step, analogous to that in laparoscopic cholecystectomy. Finally, they drafted a proposal for a laparoscopic standard technique that provides procedural safety and radicality.

To develop the proposal, the group first individually reviewed expert videos of laparoscopic CME right hemicolectomies to identify essential and potentially difficult steps of the operation. Then, they participated in a wet lab workshop evaluating each of the variants in vascular anatomy, surgeon preferences for performing the procedure, and validating the key steps of the operation previously identified. An anatomist oversaw the operations in the wet lab. A meticulous protocol was used, where the procedures were interrupted and discussed after each surgical step as well as after any complications or unforeseen difficulties. At the end of the workshop, consensus was reached regarding the procedural step sequence, representative anatomical scenes, and critical views needed for a universal standardized procedure. A final meeting reviewed videos of laparoscopic right hemicolectomies after implementation of the previous consensus to make any adjustments and finalize the definitions, relevant anatomy, proposed steps, and critical views.

The culmination of the group consensus was nine detailed steps with eight corresponding critical views to facilitate the radical procedure safely and by proper oncologic principles.

The paper is a top 10 article because it provides an invaluable standardized model for safely performing a new and difficult procedure. The operation is segmented in great detail with pitfalls for both the trainer and trainee identified. This work is unique in the painstaking detail the authors dedicated to each step- objectively defining the steps of the operation, validating the sequence with anatomic dissections and video review, and finalizing a standardized procedure with associated critical views. Each step and each critical view as well as its sequence within the procedure were discussed until full consensus was reached. It is important to read this paper before prior to attempting CME because the work serves as a procedural atlas for CME. It details the relevant definitions and surgical anatomy for appropriately performing the radical oncologic resection and provides a step-by-step description with the open-book embryologic plane model, intraoperative critical views of safety, and videos for each step, which may contribute to safe implementation of this procedure.

While this is a recent paper, it is already highly cited and referenced for programs and individuals performing CME. It will have a great impact on the field, for programs and individuals teaching and training on the CME procedure. It will also have an impact in the design of future protocols for implementing new procedures. This protocol enabled the surgical expertise of all participants on the progression of the procedure to have maximal effect. The consensus approach for defining relevant surgical anatomy, how the operation should be performed, and identifying critical views of safety may become a prerequisite for future implementation of other procedures.

There are many strengths in this work. A multidisciplinary team was established for the consensus. A large group of experienced surgeons together with an anatomist were able to find a consensus about a putative safe way to perform and teach laparoscopic right hemicolectomy with CME. The protocol offers detailed step-by-step illustrations and supplemental videos for each step, presenting multiple options for the learner. The authors also used critical views independent of surgical devices, surgical approach, or dissection techniques in the standardization so surgeons can use the tools they feel most comfortable with when following the standardized principles. There are weaknesses to consider. The expert opinion does not constitute evidence that the standardized proposed procedure is safer in practice than any other approach to laparoscopic right hemicolectomy with CME. Furthermore, since evaluation of CME quality was not the objective of the protocol, no tissue samples were histologically analyzed for the proof of oncologic superiority to standard approaches.

In conclusion, this work offers a standardized approach for laparoscopic right hemicolectomy with CME based on the open-book model and using the critical views of safety which may contribute to a safe implementation of this procedure in routine practice.

### Lee SJ et al (2016) Vascular anatomy in laparoscopic colectomy for right colon cancer [12]

This manuscript covers the important topic of the variability in vascular anatomy encountered during oncologic right colon resection for colon cancer, specifically when complete mesocolic excision (CME) with central vascular ligation (CVL) is performed.

The aim of the manuscript was to describe vascular variations around the CGT, middle colic, and ileocolic vessels to facilitate safe completion of oncologic resection during laparoscopic resection of right-sided colon cancers. Based on retrospective review of intraoperative vascular anatomy during laparoscopic right colectomy, the authors could classify variations in venous anatomy based on 1. the presence or not of a GCT (confluence of the right gastroepiploic vein, superior (or accessory) right colic vein and anterior superior pancreaticoduodenal vein),and several GCT configurations, 2. tributaries of the SMV, and 3. relationship of the ileocolic and right colic artery to the SMV. This simple classification can assist surgeons with correct identification of the vascular anatomy near the SMV, improve the quality of oncologic resections, and reduce the risk of bleeding.

A total of 116 consecutive patients with right-sided colon cancer undergoing laparoscopic right colectomy using the cranial-to-caudal approach at a single tertiary referral center were evaluated. Photographs and videos of the vascular anatomy during these cases were reviewed retrospectively and analyzed by 3 surgeons.

Venous variations around the GCT were classified into type I (presence of GCT, in 79.2%) and type II (absence of GCT, i.e., no common trunk between the colic and gastroepiploic vein, in 20.7%). Type I includes 3 subtypes based on the presence and location of the accessory superior right colic vein, and type II includes 3 subtypes based on the presence and location of a colic vein. In investigating the tributaries of the SMV, one, two, and three middle colic veins were found in 74.1%, 22.4%, and 3.5% of patients, respectively. Although a superior (or accessory) right colic vein was identified in 83.6% of patients, a right colic vein was only identified in 19%, but always drained directly into the SMV. All patients were found to have a single ileocolic vein draining into the SMV and a single ileocolic artery originating from the superior mesenteric artery (SMA). A right colic artery was identified originating from the SMA in 32.7% and patients, and coursed anterior or posterior to the SMA in 50% of patients, respectively.

This manuscript provides a detailed outline of vascular variations with diagrams to better understand the pertinent anatomy during laparoscopic right hemicolectomy. The manuscript is limited by the fact that the authors could not identify the complete paths of venous drainage of the right colon. Finally, the intraoperative ability to trace and identify all the tributaries to the venous confluence intraoperatively was not correlated to clinical outcomes such as number of lymph node harvested, short-, or long- term oncologic outcomes.

Most prior reports delineating variations in vascular anatomy have used cadaver dissections alone or in combination with radiologic imaging. This manuscript uses intraoperative findings during a cranial-to-caudal approach to laparoscopic right hemicolectomy with CVL to map out the vascular anatomy encountered near the pancreas and facilitate dissection along the superior mesenteric vein (SMV). Along with the descriptive diagrams included, this manuscript serves as a useful guide to the vascular anatomy encountered during laparoscopic right hemicolectomy for cancer.

Thorough knowledge and understanding of the variations in the gastrocolic trunk of Henle (GCT) and tributaries of the SMV, as well as variable relationships between the ileocolic, middle colic, and superior mesenteric vessels, are critical to safely complete lymph node dissection and lower the risk of bleeding complications during oncologic resection for right colon cancer.

This descriptive report on the vascular anatomy and common variations encountered during laparoscopic right hemicolectomy with CVL for cancer provides useful anatomic guidance to complete oncologic resections and avoid vascular injury.

### Cabot JC et al (2010) Long-term consequences of not closing the mesenteric defect after laparoscopic right colectomy [13]

This manuscript addresses a common dilemma faced by surgeons during laparoscopic right colectomy, namely whether leaving mesenteric defects open negatively impacts outcomes.

Leaving the mesenteric defect created open, while routine after open surgery, has been speculated to increase the risk of postoperative bowel obstruction after laparoscopic right colectomy. Prior to this manuscript, the question had not been investigated beyond scattered case reports.

With adoption of laparoscopic right colectomy, it is important to weigh the challenge of closing the mesenteric defect against risks associated with leaving an open defect. Laparoscopic closure of the mesenteric defect can be technically challenging with risk of bleeding, ischemia to the anastomosis, and small bowel incarceration through an incompletely closed small mesenteric defect.

This paper addresses the long-term safety of a laparoscopic approach for right colectomy with respect to management of the mesenteric defect.

In this retrospective review, the long-term safety of laparoscopic right colectomy over a 7-year period by 9 surgeons was evaluated.

Among 530 consecutive cases, 6.8% were converted to open. At a median follow-up of 20 months, 26 patients (4.9%) developed small bowel obstruction, 12 of which within the first 30 days and 21 within the first year of surgery. Fourteen patients (54%) required operative intervention including 4 cases in which the cause of obstruction was attributed to the mesenteric defect (0.8% of the entire cohort). Two of these cases involved torsion of the anastomosis through the mesenteric defect and two were due to internal herniation of the small bowel through the defect. Three of these 4 cases presented with SBO within 30 days of colectomy while the 4^th^ presented 8 months postoperatively, and all were corrected without closure of the mesenteric defect. The only statistically significant difference between the groups was the higher proportion of males in the group who developed SBO.

This relatively large series permitted long-term assessment of the relative incidence of mesenteric defect-related complications following laparoscopic right colectomy. Limitations of the manuscript include its retrospective design, the variable follow-up interval, and the lack of a comparison group. Finally, it is not possible to infer the definite cause of obstruction in patients who were managed without surgery, which may have underestimated the true the incidence of internal hernia.

Overall, this manuscript concluded that complications related to the open mesenteric defect in right colectomy are rare. The current standard practice of leaving the mesenteric defect open is safe with respect to the risk of internal hernia and avoids the technical challenges of closing the defect laparoscopically. The rate of small bowel obstruction from leaving the mesenteric defect open is low, and bowel obstruction more commonly results from other issues, including twisting of the anastomosis. The authors suggest re-insufflating the abdomen after completion of extracorporeal anastomosis to confirm correct orientation of the bowel limbs and reduce the risk of twisting of the anastomosis through the mesenteric defect.

### van Oostendorp S et al (2017) Intracorporeal versus extracorporeal anastomosis in right hemicolectomy: a systematic review and meta-analysis [14]

This manuscript addresses a controversial topic related to the choice of anastomotic reconstruction during laparoscopic right colectomy. It provides a systematic review of outcomes following intracorporeal (IA) versus extracorporeal anastomosis (EA) during laparoscopic right colectomy. It includes all identified studies that compared perioperative mortality, morbidity, and length of hospital stay between these two techniques.

The Preferred Reporting Items for Systematic Reviews and Meta-Analysis (PRISMA) guidelines were used and the search was performed on PubMed and Embase. Studies eligible for inclusion consisted in randomized controlled trials and comparative studies on intra- versus extracorporeal anastomosis during laparoscopic right hemicolectomy. Studies with single-incision, robotic surgery, and open colectomy cohorts were excluded. Two independent reviewers selected the studies and analyzed the resulting papers in full text using the online Covidence review manager (www.covidence.org). The MINORS instrument (methodological index for nonrandomized studies) was used to assess methodological quality of the included studies.

A total of 12 comparative studies were selected for meta-analysis including one prospective and 11 retrospective studies, encompassing 1,492 patients undergoing elective laparoscopic right hemicolectomy with extracorporeal (*N* = 729) and intracorporeal anastomosis. IA was associated with decreased length of stay (MD − 0.77 days, 95% CI − 1.46 to − 0.07) and decreased rates of surgical site infections (OR 0.56, 95% CI 0.35–0.88) with no differences in anastomotic leaks (OR 0.77, 95% CI 0.39 – 1.49) or ileus (OR 0.94, 95% CI 0.57 − 1.57). Operative times differed widely across studies with most reporting no significant differences between the groups.

The meta-analysis is limited by the fact that of the 12 studies included, only one study was prospective and no randomized trials existed to include at the time of publication. The data included were mostly retrospective with substantial heterogeneity in the reporting of postoperative morbidity.

Overall, this systematic review comparing intracorporeal and extracorporeal anastomosis during laparoscopic right hemicolectomy shows that the IA technique is associated with significant decrease in the rate of surgical site infection and length of hospital stay. There were no differences in mortality, ileus, and anastomotic leakage rates. The improved outcomes associated with the IA technique may be primarily related to the extraction site since Pfannenstiel incisions have been shown to reduce incisional pain and infection rate relative to periumbilical incisions. However, other factors associated with IA may account for these differences in outcomes and warrant further investigation. A randomized controlled trial may be helpful in providing further evidence to support wide adoption of IA into surgical practice.

This is the largest and most current systematic review and meta-analysis of all publications comparing both techniques that encompass a large volume of cases for robust comparison between the two groups.

Many surgeons have been reluctant to adopt intracorporeal anastomosis in the setting of laparoscopic right hemicolectomy due to lack of definitive evidence of clinical benefit relative to extracorporeal anastomosis. This meta-analysis may provide such evidence and alter practice. Surgeons may consider changing their current practice based on this manuscript’s findings.

### Tekkis PP et al (2005) Evaluation of the learning curve in laparoscopic colorectal surgery: comparison of right-sided and left-sided resections [15]

This manuscript documents the learning curve of laparoscopic colorectal surgery. At the time of its publication, the learning curve was unknown. The contemporary COST laparoscopic vs. open colon cancer trial had a relatively arbitrary threshold of 20 cases for surgeon credentialing. This trial not only helped clarify the learning curve and risk factors for difficult and challenging cases, but it also tracked as one of its primary outcomes of patient-centered safety data of complications and readmission rates.

This study is a risk adjusted, multidimensional analysis of the learning curve of laparoscopic colorectal surgery examining 900 cases over 12 years at a single academic institution. The authors chose to track both surgeon-specific outcomes (conversion rates and operative times) and patient-specific outcomes (complication and readmission rates) for two procedures: left- and right-sided colectomy. The primary outcome, conversion rate, was analyzed using risk adjusted cumulative sum (CUSUM). Conversion was defined as early or unplanned conversion to open or creation of an extraction site > 10 cm.

Surgeon-specific outcomes demonstrated learning curves and conversion rates of 55 cases and 8.1%, respectively, for right-sided colectomy and 62 cases and 15.3%, respectively, for left-sided colectomy. Ninety-nine percent of the study cases were completed by 4 surgeons who performed 348, 310, 136, and 95 cases, respectively. Independent risk factors for conversion included intraabdominal abscess (OR 5), intraabdominal fistula (OR 4.6), ASA (OR 1.63 per unit increase), BMI (OR 1.07 per unit increase), left colectomy (OR 1.1), and surgeon operative experience (OR 0.9 per 10 cases). Median operative times for the first 25 cases were 180 min and decreased to 115 min at the end of the series (> 175 cases), but did not differ between left- and right-sided resections. Patient-specific outcomes were similar between the two groups and remained similar over the learning curve. Readmission and complication rates were 10.5% and 20.8% for right colon resections, and 6.2% and 17.8% for left. Multifactorial logistic regression analysis of risk factors for complications showed two independent factors: ASA (OR 1.4 per unit increase) and conversion to open surgery (OR 1.7). Significant risk factors for readmission were left-sided resection (OR 0.47), prior abdominal surgery (OR 2.2), and intraabdominal fistula (OR 3.9).

Inclusion of a mature case series of early adopters of laparoscopic colorectal surgery and use of risk-adjusted CUSUM to analyze the primary outcome are strengths of the manuscript. The limitations are a relatively small number of study subjects for 4 surgeons. The evolving surgeon experience results in clinical heterogeneity both in patient selection and complexity of procedures over the course of the study. Further, the authors do not specifically state the involvement of trainees during these cases and their possible effect on study outcomes, particularly operative times.

The initial learning curve for a laparoscopic right hemicolectomy is 55 cases. This number can be affected by ASA and conversion to open surgery. As surgeons gain experience and confidence, they naturally become less restrictive in their patient selection criteria and complexity of procedure performed laparoscopically. This manuscript provides important guidance to understand the learning curve required for this complex operation, and risk factors that make the laparoscopic approach more technically challenging.

This manuscript reinforces the fact that laparoscopic colorectal surgery is challenging, is not for every patient, and that a surgeons’ skill set develops over time.

## Conclusion

Competence in laparoscopic right colectomy can be learned from a structured educational curriculum coupled with focused training and practice. An essential first step in the learning process is pre-training, where the surgeon familiarizes themselves with the anatomy, technical steps, and clinical pearls prior to adopting the procedure. The SAGES Masters program is intended to improve the knowledge and skills of practicing surgeons and increase safe adoption of minimally invasive procedures. Among the didactic resources included in the laparoscopic right colectomy pathway, the top 10 seminal articles selected encompass the best evidence for this approach and the most impactful technical descriptions for safe performance for both benign and malignant diseases. This list will be updated regularly to assure that the best evidence and learning experience is provided for surgeons completing the SAGES Masters Program.
